# Peer Tutoring Effects on Students’ Mathematics Anxiety: A Middle School Experience

**DOI:** 10.3389/fpsyg.2020.01610

**Published:** 2020-07-29

**Authors:** Lidón Moliner, Francisco Alegre

**Affiliations:** ^1^Department of Education, Jaume I University, Castellón de la Plana, Spain; ^2^Department of Didactics of Mathematics, University of Valladolid, Valladolid, Spain

**Keywords:** peer tutoring, mathematics anxiety, middle school, reciprocal tutoring, effect size, mixed methods, learning anxiety, evaluation anxiety

## Abstract

In this research the effects of reciprocal peer tutoring on students’ mathematics anxiety levels were examined. A pretest posttest with control group design was used at a public middle school in Spain. A total of 420 students in 7th, 8th, and 9th grades participated in the study, of which 215 were female and 205 were male. Students were randomly assigned and equally distributed by course grade (140 in each course grade) and experimental condition (210 in the experimental group and 210 in the control group). Quantitative data were gathered using the Mathematics Anxiety Scale developed by Chiu and Henry (1990). Qualitative information was gathered during eight focus group sessions that were held with students. Two main factors were analyzed using the quantitative and qualitative information: mathematics learning anxiety and mathematics evaluation anxiety. Results were analyzed by gender and course grade. Statistically significant improvements were reported for both male and female students in the experimental group and for each course grade for both factors. No statistically significant differences were reported for students in the control group in any case. A moderate effect size was reported for mathematics evaluation anxiety (Hedge’s *g* = 0.42), and a large effect size was reported for mathematics learning anxiety (Hedge’s *g* = 0.84). Information obtained from the focus groups was consistent with the reported quantitative results. The main conclusion is that peer tutoring may be very beneficial for reducing middle school students’ mathematics anxiety, regardless of their gender or grade.

## Introduction

### State of the Problem and Need for This Research Study

Authors such as Passolunghi et al. (2016), Foley et al. (2017), and Núñez-Peña and Bono (2019) recently addressed the link between mathematics anxiety and mathematics achievement among secondary education students. According to them, mathematics anxiety has a significantly negative impact on students’ achievement in mathematics. Several authors in the educational psychology field, including Holmes and Hwang (2016), Guita and Tan (2018), and Choi-Koh and Ryoo (2019), found that cooperative and active learning methodologies may decrease students’ mathematics anxiety and, as a result, positively impact their academic performance in mathematics. This finding has been long supported by authors like Stodolsky (1985), who attributed students’ high levels of mathematics anxiety to a lack of social support provided through cooperative learning strategies such as peer tutoring. Peer tutoring is one of the learning methodologies that has been studied the most in the field of cooperative learning. Indeed, authors such as Topping (Topping and Whiteley, 1993; Shanahan et al., 1994; Topping et al., 2011; Topping, 2019), Fuchs and Fuchs (Fuchs et al., 1995, 2019; Powell and Fuchs, 2015), and Ginsburg-Block and Fantuzzo (Fantuzzo and Ginsburg-Block, 1998; Ginsburg-Block et al., 2006; Can and Ginsburg-Block, 2013), among others, have been studying the academic, social, and psychological benefits of peer tutoring in mathematics and other subjects for more than three decades. The positive effects of this cooperative learning strategy on variables such as self-concept, attitude toward mathematics, self-esteem, and social integration have been repeatedly documented (Alegre et al., 2020; Moliner and Alegre, 2020). Nevertheless, in spite of the broad range of literature that exists regarding peer tutoring, very few studies have addressed the effects of this methodology on students’ mathematics anxiety. Studies by Reyes and Castillo (2015) and Garba et al. (2019) have shown promising results but are limited in terms of information, and both suggest further research on the effects of peer tutoring on students’ mathematics anxiety. Hence, given the need for students to participate in cooperative and active learning methodologies that lower their mathematics anxiety, and given the proven positive effects of peer tutoring on academic achievement and other psychological variables, a study testing the effects of peer tutoring on students’ mathematics anxiety can not only build on the existing literature, but also inform educators on best practices for helping students with mathematics anxiety to improve their performance.

### Mathematics Learning Anxiety vs Mathematics Evaluation Anxiety

In this research two main constructs are analyzed: mathematics learning anxiety and mathematics evaluation anxiety. On one hand, mathematics learning anxiety may be defined as feelings of fear, tension, and apprehension that some people feel during the study and assimilation of mathematics contents (Powell et al., 2019). Authors such as Lazarides and Buchholz (2019) consider that students must control this type of anxiety and highlight its importance as a prerequisite for academic outcome in mathematics and well-being. On the other hand, mathematics evaluation anxiety may be defined as worry brought on by examinations and tests or other evaluation of performance in mathematics (Everingham et al., 2017). Authors such as Lu et al. (2019) highlight its importance as they state that this type of anxiety may be developed even from the earliest years of mathematics instruction in kindergarten. The differences and relationships between these two types of mathematics anxiety has been addressed recently. In this sense, authors such as Schillinger et al. (2018) state that although evaluation anxiety and lerning anxiety have shared variance, they may also be thought of as separable constructs. Authors such as Pizzie and Kraemer (2017) consider that both types of anxiety are highly correlated, play a vital role in students’ performance in mathematics and that must be studied in depth.

### Gender and Age Differences Regarding Mathematics Anxiety

The effectiveness of an academic intervention in psychological variables may be influenced by variables such as students’ gender or age. In this sense, previous studies have shown important differences between female and male students regarding mathematics students. Research by Karimi and Venkatesan (2009), Ganley and Vasilyeva (2014), or Stoet et al. (2016) reported significant gender differences in mathematics anxiety in different academic interventions. These authors highlight the importance of analyzing the effects separately and altogether when studying mathematics anxiety. Analogously, authors such as Baloglu and Kocak (2006) or Sidney et al. (2019) state that differences in mathematics anxiety may be reported even within the same educational levels. One of the main conclusion of their studies is that both, age and gender differences should be investigated in the studies of mathematics anxiety and that the multidimensionality of anxiety should be carefully taken into account.

### Peer Tutoring: Conceptual Framework

Peer tutoring may be defined as a cooperative and active learning strategy in which students help each other in dyads, while learning at the same time (Alegre Ansuategui and Moliner Miravet, 2017). Zapata (2020) noted that students of different educational levels have very positive perceptions of this learning methodology. Different types of peer tutoring may be implemented, depending on students’ abilities, academic competencies, organizational issues, material, and personal resources. Traditionally during peer tutoring, the most academically competent student serves as the tutor, and the least academically competent student serves as the tutee. When the students do not switch roles during the peer tutoring program, that is, in each pair the tutor is always the tutor and the tutee is always the tutee, the learning method is called fixed peer tutoring. When the students do exchange roles, that is, when the students go from being the tutor to being the tutee and vice versa, depending on the peer tutoring session, then the tutoring method is referred to as reciprocal peer tutoring (Youde, 2020). Moreover, peer tutoring methods may be classified according to the age of the participants: same-age peer tutoring involves a pair of students who are of the same age, while cross-age tutoring involves students of different ages (Zendler and Greiner, 2020). The benefits of peer tutoring have been documented for different subjects and at different educational levels. These benefits are not restricted to competent or proficient students, as struggling, learning disabled, and at-risk learners have also been found to benefit from peer tutoring (Huber and Carter, 2019; Mahoney, 2019; Sarid et al., 2020). Although most of the research in the field has been carried out at the primary and secondary education levels, several recent studies have focused on peer tutoring in higher and continued education (Struk et al., 2019; Ellis and Gershenson, 2020). The variety of tutoring typologies and the different organizational possibilities (for example, duration of the peer tutoring sessions, duration of the peer tutoring program, and number of sessions per week) make this learning method adaptable to different educational contexts, independent of time available for implementation and the students’ educational stages and academic competencies or abilities.

### Peer Tutoring in Mathematics: Academic and Psychological Effects

From an academic perspective, the effects of peer tutoring on students’ mathematics achievement seem to be moderate. Alegre-Ansuategui et al. (2018) performed a meta-analysis on peer tutoring and academic achievement in mathematics. The reported average effect size was moderate, and most studies included in the meta-analysis reported statistically significant improvements. The authors who conducted the meta-analysis noted that peer tutoring interventions in primary education seemed to be more effective than those implemented in secondary education. This difference may also be appreciated when considering the results of the meta-analytic reviews conducted in primary education (Alegre et al., 2019a) and secondary education (Alegre et al., 2019b). Although the reported average effect size was moderate in both reviews, it was somewhat larger for the primary education study than for the research that focused on secondary education.

From a psychological perspective, mathematics self-concept is the primary variable that has been analyzed through the years. Studies conducted by Fantuzzo et al. (1995), Lee and Park (2000), Topping et al. (2003), Tsuei (2012), Zeneli et al. (2016a), and Alegre Ansuategui and Moliner Miravet (2017) consistently reported significant improvements in students’ mathematical self-concepts as a result of peer tutoring. Various social, behavioral, and academic meta-analyses in the peer tutoring field all revealed that significant improvements may be found from a psychological perspective when this learning methodology is implemented (Leung et al., 2005; Ginsburg-Block et al., 2006; Bowman-Perrott et al., 2013, 2014).

## Materials and Methods

The Valencian Ministry of Education institutional review board authorized this research. The board approved the research, but the consent obtained specified that data had to be analyzed anonymously.

### Aim of the Study and Hypotheses

The main aim of this research was to determine the effect of peer tutoring on middle school students’ mathematics anxiety. To this purpose, as stated above, two main factors were analyzed: mathematics learning anxiety and mathematics evaluation anxiety. Considering the aim and the analyzed factors, the following three hypotheses were defined.

First, as indicated in the introduction section, significant statistical improvements and moderate effect sizes may be expected when implementing peer tutoring and targeting psychological variables. Hence, hypothesis 1 and 2 were defined as follows.

Hypothesis 1: Statistically significant differences will be reported between the pretest and the posttest for students in the experimental group in both, mathematics learning anxiety and mathematics evaluation anxiety and moderate effect sizes will be reported.
Hypothesis 2: Posttest scores for the experimental group in both, mathematics learning anxiety and mathematics evaluation anxiety will be significantly lower than the posttest scores for the control group.

Moreover, as previously stated, several authors highlight the importance of addressing age and gender differences in mathematics anxiety studies. Hence, given this fact, hypothesis 3 and hypothesis 4 were defined as follows.

Hypothesis 3: No statistically significant differences will be reported for the posttest scores among 7th, 8th, and 9th grade students’ in the experimental group in mathematics learning anxiety or mathematics evaluation anxiety.
Hypothesis 4: No statistically significant differences will be reported for the pretest or posttest scores between female and male students’ mathematics learning anxiety and mathematics evaluation anxiety.

### Research Design

Authors such as Zeneli et al. (2016b) and Alegre et al. (2019a) have highly recommended including control groups when conducting peer tutoring studies in middle school mathematics, noting that the absence of a control group may result in an overestimation of the effect sizes resulting from the study. Hence, following the guidance provided by these authors, a quasi-experimental pretest posttest with control group design was used in this research (Nind and Lewthwaite, 2019).

### Sample Access

Weaver and Snaza (2017) and Chen and Reeves (2019) addressed the difficulty in obtaining a proper sample for educational studies. Participants in this research were selected intentional sampling, that is, non-probabilistic sampling technique (Yue and Xu, 2019). One public middle school in Spain was selected for this research after researchers suggested it to the Valencian Educational Government. Written and informed consent was obtained from the parents or guardians of students who participated in the study. Written authorization was also obtained by the School Council and the Valencian Educational Government. Research ethics provided by the Ethics Committee of the Spanish National Research Council (CSIC) were followed during the study.

### Participants

A total of 420 students from grades 7–9 participated in the research. Their ages ranged from 12 to 15 years old. The mean age at the beginning of the study was 13.56 years old with a standard deviation of 1.25 years, and the median value was 13.67. Students were equally distributed by course grade, that is, there were 140 students from each of the three participating grade levels. Further, 215 (51.19%) were female, and 205 (48.81%) were male, while 223 (53.10%) were Hispanic, 99 (23.57%) were Rumanian, 68 (16.19%) were African, 5 (1.19%) were Asian, and the other 5.95% were from other ethnic groups. The students were from families of average sociocultural and socioeconomic status, according to national standards. Students were assigned to the experimental or the control group following as follows. Class groups were already established at the beginning of the course. Half of the class groups in each grade were randomly allocated to experimental conditions and the other half acted as control group in each grade. Therefore, half of the students from each grade were randomly allocated to the experimental group and the remaining half to the control group.

### Sample Power

StudySize 3.0 software by Creostat HB was used to determine the sample power. A sample power of 0.92 was determined when using inferential statistics (Students’ *t*-test and Analysis of Variance) with a significance level of 0.05 for 420 participants.

### Peer Tutoring Intervention

#### Academic Content

The mathematical content worked on by the students during the peer tutoring implementation included algebra, geometry, statistics, and probability. This content corresponded to the second and third trimesters of the math courses for each grade. Seventh grade students worked with basic first degree equations, used the Pythagorean theorem, calculated surface areas and regular prism volumes, calculated basic statistical centralization parameters for qualitative and quantitative variables, used the Laplace rule, and completed basic tree diagrams for probability problems. Eighth grade students updated the course content of the previous year as described above and also calculated compound probabilities, standard deviations and variations, and first-degree equations with fractions; performed basic systems of equations; and calculated the volumes of irregular prisms. The ninth grade students also updated the previous content and worked with quartiles, percentiles and box diagrams; developed advanced tree diagrams; applied the Laplace succession rule; calculated complex surfaces and volumes; performed complex systems of equations; and solved third and fourth direct resolution degree equations (using Ruffini’s rule and factorization).

#### Typology of the Peer Tutoring Intervention

The same-age, reciprocal peer tutoring method was used in this research. This type was selected over other types (cross-age or fixed) for different reasons. First, cross-age tutoring is more complicated than same-age tutoring to implement in middle school settings (Alegre et al., 2019b) for organizational and scheduling reasons, as arranging for students of different ages and from different grades to meet for tutoring sessions can be challenging due to the different schedules followed by the different grades. Moreover, cross-age tutoring most often occurs with the elder student tutoring the youngest student; that is, employing fixed peer tutoring is almost a must for cross-age tutoring. Therefore, cross-age was absolutely discarded as an option. Further, several authors point to reciprocal peer tutoring as providing greater benefits for psychological variables than fixed tutoring (Moeyaert et al., 2019; Sytsma et al., 2019), which they attributed to the students’ exchanging tutor and tutee roles, which does not happen during fixed peer tutoring. Hence, tutees may feel less competent or not as useful as their peers (Gazula et al., 2017). Thus, same-age, reciprocal peer tutoring was deemed most appropriate for this study.

#### Organization and Scheduling

During the first trimester of the school year, mathematics teachers in all classes used traditional teaching methods. Students sat individually, interactions between them were limited, and the one-way instructional teaching method was employed. All students participating in the study took the pretest right after the first trimester ended. Then, during the second and third trimesters peer tutoring was implemented. Students in the experimental group worked through peer tutoring in their mathematics classes, while students in the control group continued with the one-way traditional learning methods above mentioned (but did not participate in peer tutoring). Students in the control group sat individually and interactions between them were restricted. Students in both, experimental and control group, had the same teacher in each grade. Students in the experimental and control groups were given the same exercises and problems for every session. If a pair of students in the experimental group solved the task correctly, although tutoring was not necessary in these occasions, they were told to share the procedures they had employed to solve the exercises or problems.

In order to maximize the psychological outcomes of the peer tutoring intervention, the organizational issues for this research followed the structure provided by Rees et al. (2016) and Leung (2019a, b). As such, peer tutoring was implemented three times per week for 6 months with students in the experimental group. Interaction between peers lasted no more than 20 min. The same exercises and problems were given to students in both the experimental group and the control group throughout the year in each grade, and both groups used the same type of materials (textbook, worksheets, and online exercises, for example). Moreover, the same teachers taught students in both groups so that teacher effects did not influence the psychological outcomes (Cleary and Kitsantas, 2017).

Distribution of pairs was carried out following the indications by Duran (2017). According to this author, variations in students’ academic achievements must be minimized for students placed in pairs for reciprocal peer tutoring. Hence, in order to arrange the pairs, students were placed from highest to lowest, taking their average mathematics mark of the first trimester. In other words, the first student, that is, the student at the top of the list, was paired with the second student (the student with the second highest score or grade), and then the third was paired with the fourth, and so on. Several authors note that students prefer this way of pairing because they are assigned to work with a peer whose competency in that subject is similar to theirs (Thurston et al., 2019).

#### Students’ Peer Tutoring Training

Students in the experimental group were trained in two sessions of 1 h each on tutoring skills and procedures the week before the peer tutoring program began. They took place during school hours to ensure students’ attendance. This training was carried out by the same mathematics teachers who taught the students during the year. Although the teachers conducted these sessions, students also participated actively. For example, students were asked to identify those characteristics and qualities that good tutors and good tutees must have to succeed in peer tutoring. In addition, students were instructed on the procedure to follow during the tutoring sessions and on the nature of their interactions. They were given “Pause, Prompt, and Praise” techniques and were advised on the importance of communication during the tutoring sessions (Duran et al., 2019a). Issues like sharing only mathematics content, referring only to the mathematics exercises and problems, and not talking about other non-academic subjects during the peer tutoring sessions were highlighted. Different ways to explain content to a peer and different procedures employed to solve a problem were praised. Patience and respect were emphasized, and a main goal was defined for the tutoring sessions: all students had to understand and finish the exercises and problems by the time the tutoring session was over.

#### Classroom Dynamics During Peer Tutoring

The dynamics of the classroom were as follows. First, the teacher reviewed the students’ homework, provided the correct answers on the board, and explained the new content, all of which took about 20 min. After that, students had to complete two exercises and one or two problems, depending on the difficulty of the didactic unit. Students were given approximately 15 min to complete these tasks and were instructed to complete the tasks individually, without interacting with their classmates. During this time, the teacher could help students who didn’t know how to complete the exercise or solve a problem. At this point, the teacher also checked to make sure that at least one of the two students in each pair had solved the exercises and problems correctly. If this was not the case, the teacher provided assistance. Afterward, the students participated in the reciprocal peer tutoring sessions for approximately 20 min to check and finalize the work they had done individually. Indications and protocols analogous to those provided by Moliner and Alegre (2020) were followed during peer tutoring. Working in pairs, students had to compare the results they had arrived at when working on their own, share the procedures they had employed to solve the tasks, ask each other questions regarding the exercises and problems, and work together to solve any problems that they hadn’t completed when working independently. If they had different results for any of the work, both tutor and tutee had to try to identify the mistake at the same time. Then the student with the right answer had to help the other student by explaining how to correctly solve the problem. Students were allowed to ask questions regarding the exercises and problems and help each other during tutoring, but individual work and perseverance were a must. Both tutors and tutees had to be able to solve the exercises and problems by themselves by the time the tutoring period was over. If a pair of students finished their work very early, they were given additional problems. When the tutoring session was over, for the last 10 min of class, the teacher provided and explained the correct answers to the exercises and problems on the board.

Interactions between pairs of students were supervised by the teacher. As Duran et al. (2019b) stated, teachers play a vital role during the implementation of peer tutoring. They must ensure that communication between students is respectful and rich in content and that students are effectively working together and helping one another.

### Instrument Used to Collect Information

Students’ mathematics anxiety was measured using the Mathematics Anxiety Scale developed by Chiu and Henry (1990). This instrument is based on a 4-point Likert scale with no reversed items. Students were asked to rate each item to document how they felt according to the following scale: 1 (not nervous), 2 (a little bit nervous), 3 (nervous), and 4 (very nervous). The average score indicated students’ anxiety level in mathematics. The higher the average score, the higher the student’s mathematics anxiety level. Two main factors were defined in the questionnaire: mathematics learning anxiety and mathematics evaluation anxiety. The mathematics learning anxiety factor was assessed by six items, such as (item 5) starting a new chapter in a mathematics book or (item 6) watching a teacher work a mathematics problem on the chalkboard. The mathematics evaluation anxiety factor was assessed using eight items, such as (item 10) thinking about a math test the day before the test or (item 12) taking an important test in a mathematics class. This instrument was selected because it is specifically geared toward middle school mathematics students, because its psychometric properties, validity, and reliability have been repeatedly documented (Beasley et al., 2001; Lukowski et al., 2019), and because it has been widely used for decades and continues to be used in the field of educational psychology (Fan et al., 2019; Namkung et al., 2019; Van Mier et al., 2019). The average scores for each of the two factors were calculated and used as measures of students’ mathematics anxiety for use in this study. Students completed the questionnaire individually during tutoring time. It took less than 10 min for almost all students to complete it. Researchers explained to the students how to complete the questionnaire and remained with them while they completed it to answer questions. As the instrument was originally designed in English, each item was translated to Spanish and adapted to the Spanish population by a professional translator. A reliability analysis was performed with SPSS software version 25 to ensure that the psychometrics properties of the instrument had not been significantly altered for this research. The pretest scores for students in both, experimental and control group were used to perform this analysis. A Cronbach’s alpha value of 0.91 was reported for Mathematics learning anxiety factor and a Cronbach’s alpha value of 0.93 was reported was mathematics evaluation anxiety factor. These values were almost identical to the original values reported by Chiu and Henry (1990).

Focus groups were used to collect qualitative information from the students (Carter Andrews and Gutwein, 2020). A total of 28 students (7 focus groups of 4 students each) from the experimental group were randomly selected to participate. The protocol was as follows: a draw was performed including students’ of all grades until 28 students were selected. The first four students selected constituted the first focus group, the next four the second group and so on. Students were told that they had been randomly selected and were asked individually if they wanted to participate in the focus group. Two of the researchers conducted the focus groups (both were present in each of them). The questions asked by the researchers during these focus groups were aimed directly at revealing the anxious feelings students experienced during peer tutoring (Bokhorst-Heng and Marshall, 2019). Specifically, the students’ feelings about learning anxiety and evaluation anxiety were addressed through questions such as “Why do you think that you feel more or less stressed during mathematics classes?” or “How did you feel during the exam after peer tutoring?” These focus group sessions, lasting about 20 min each, were held during tutoring hours in private spaces.

In order to avoid any Hawthorne effect (Greener, 2018), students were not told that research was being conducted or that they were taking part in a study. They were not told they belonged to a experimental or control group. This was done to try that students did not modify their behavior or alter their answers in the questionnaires or during the focus group sessions as a result of being aware that they were being observed (van Alten et al., 2019).

### Data Analyses

Quantitative data coming from the Mathematics Anxiety Scale was analyzed using SPSS software version 25. The Kolmogorov Smirnov test was performed to ensure normality of the data for the pretest scores in the experimental and control groups (Fang and Chen, 2019). Means, standard deviations, and Student’s *t*-test (95% confidence level) were calculated for both mathematics learning anxiety and mathematics evaluation anxiety in order to determine differences between and within groups (Gibbs et al., 2017). Analyses of variance (ANOVAs) were also performed to identify differences among 7th, 8th, and 9th grade students. Given the fact that in this research multiple comparisons are carried out, inferential tests were performed with a notion of correcting for multiple assessments. Hence, the Bonferroni adjustment (Umlauft et al., 2019) implied that differences between and within groups would need a significance level of *p* < 0.01 instead of *p* < 0.05 so that they could be considered as significant. Effect sizes were reported for each of the two analyzed factors. Hedge’s *g* was used as a measure of effect size (Ebner and Gegenfurtner, 2019). Rule of thumb provided by Lee et al. (2019) and Morris (2019) for effect sizes was followed. According to these authors, in educational psychology the following values may be used for interpreting results. A Hedges’ *g* value of 0.2 indicates a small effect, a value of 0.5 indicates a moderate or medium effect, and a value of 0.8 or higher indicates a large effect size.

Qualitative data from the focus group sessions were analyzed using content analysis (Adler et al., 2019). ATLAS.ti software version 8 was used for this purpose. After the transcription of the conversations from the focus group sessions, researchers analyzed the information and defined two main dimensions: mathematics learning anxiety and mathematics evaluation anxiety. The students’ quotes were codified as number of focus group and grade: for example, FG2_9 refers to focus group number 2 of 9th grade.

## Results

### Quantitative Results

**FIGURE 1:**
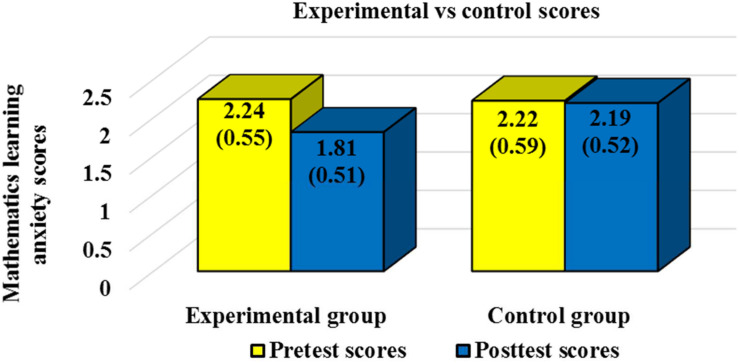
Mathematics learning anxiety pretest and posttest scores and standard deviations for the experimental and control group.

**FIGURE 2:**
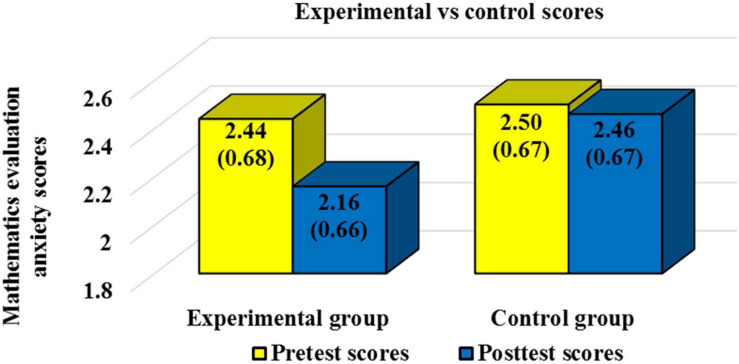
Mathematics evaluation anxiety pretest and posttest scores and standard deviations for the experimental and control group.

The Kolmogorov Smirnov test showed that students’ scores followed a normal distribution (*p* = 0.92). Means, standard deviations (SDs), and number of students (n) by group (experimental or control) and phase of the study (pretest or posttest) are shown in Table 1 for mathematics learning anxiety and in Table 2 for mathematics evaluation anxiety. In order to facilitate readers’ global vision of the results scores for the experimental and control group are represented through a graph in Figure 1 for mathematics learning anxiety and in Figure 2 for mathematics evaluation anxiety.

**TABLE 1:** Means, standard deviations and number of students by group and phase of the study for mathematics learning anxiety.

**Pretest**						**Posttest**					
**Experimental**			**Control**			**Experimental**			**Control**		
**Mean**	**SD**	***n***	**Mean**	**SD**	***n***	**Mean**	**SD**	***n***	**Mean**	**SD**	***n***
2.24	0.55	210	2.22	0.59	210	1.81	0.51	210	2.19	0.52	210

**TABLE 2:** Means, standard deviations and number of students by group and phase of the study for mathematics evaluation anxiety.

**Pretest**						**Posttest**					
**Experimental**			**Control**			**Experimental**			**Control**		
**Mean**	**SD**	***n***	**Mean**	**SD**	***n***	**Mean**	**SD**	***n***	**Mean**	**SD**	***n***
2.44	0.68	210	2.50	0.67	210	2.16	0.66	210	2.46	0.67	210

Mean differences between groups and Student’s *t*-test results are reported in Table 3 for mathematics learning anxiety and in Table 4 for mathematics evaluation anxiety. Statistically significant differences were not found between the experimental and control groups for the pretest scores. No statistically significant differences were found between the pretest and posttest scores for the control group. Statistically significant improvements were reported between the pretest and the posttest for the experimental group in both, mathematics learning anxiety and mathematics evaluation anxiety. Statistically significant differences were also reported for the posttest scores between the experimental group and the control group. In both cases, mathematics learning anxiety and mathematics evaluation anxiety experimental group posttest scores were significantly lower than control group posttest scores. A moderate effect size was reported for mathematics evaluation anxiety (Hedge’s *g* = 0.42), and a large effect size was reported for mathematics learning anxiety (Hedge’s *g* = 0.84). Therefore, hypothesis 1 (statistically significant differences will be reported between the pretest and the posttest for students in the experimental group in both, mathematics learning anxiety and mathematics evaluation anxiety and moderate effect sizes will be reported) was rejected since a large effect size was reported for mathematics learning anxiety. On the contrary, hypothesis 2 (posttest scores for the experimental group in both, mathematics learning anxiety and mathematics evaluation anxiety will be significantly lower than the posttest scores for the control group) was confirmed.

**TABLE 3:** Mean differences between groups and Students’ *t*-test for mathematics learning anxiety.

**Comparison**	**Mean difference**	***t*-value**
Experimental group pretest vs control group pretest	0.02	0.36
Control group posttest vs control group pretest	–0.03	0.33
Experimental group posttest vs experimental group pretest	–0.43	8.31*
Experimental group posttest vs control group posttest	–0.40	7.54*

*p < 0.01.

**TABLE 4:** Mean differences between groups and Students’ *t*-test for mathematics evaluation anxiety.

**Comparison**	**Mean difference**	***t*-value**
Experimental group pretest vs control group pretest	–0.10	0.91
Control group posttest vs control group pretest	–0.04	0.61
Experimental group posttest vs experimental group pretest	–0.28	4.28*
Experimental group posttest vs control group posttest	–0.34	5.30*

*p < 0.01.

ANOVAs across grades were calculated for the posttest scores of the experimental group for both, mathematics learning anxiety and mathematics evaluation anxiety. No statistical significant differences across grades were reported for mathematics learning anxiety *F*(2, 207) = 0.87, *p* = 0.42 nor mathematics evaluation anxiety *F*(2, 207) = 2.40, *p* = 0.09. Hence, hypothesis 3 (no statistically significant differences will be reported for the posttest scores among 7th, 8th, and 9th grade students’ in the experimental group in mathematics learning anxiety or mathematics evaluation anxiety) was confirmed.

The results of the analysis by gender for are reported for mathematics learning anxiety in Table 5 and for mathematics evaluation anxiety in Table 6. No statistically significant differences were reported in any case. Hence, hypothesis 4 (no statistically significant differences will be reported for the pretest or posttest scores between female and male students’ mathematics learning anxiety and mathematics evaluation anxiety) was confirmed.

**TABLE 5:** Student’s *t*-tests by gender for mathematics learning anxiety.

**Male vs female**	***t*-value**	***p***
Experimental group pretest	0.26	0.79
Experimental group posttest	0.43	0.67

**TABLE 6:** Student’s *t*-tests by gender for mathematics evaluation anxiety.

**Male vs female**	***t*-value**	***p***
Experimental group pretest	0.32	0.75
Experimental group posttest	0.66	0.51

### Qualitative Results

Information coming from the focus groups was mostly positive regarding the effects of peer tutoring on students’ mathematics anxiety. As noted in the data analysis section, this information may be classified into two dimensions: mathematics learning anxiety and mathematics evaluation anxiety. The qualitative results confirmed the quantitative information coming from the questionnaires. Regarding the first category, students’ mathematics learning anxiety seemed to have improved substantially. (All names in the following are invented for anonymity reasons.) *It’s less stressful when you have a colleague who can help you* (FG3_7). They felt less stressed when working with a peer as they had an established routine that facilitated their interactions. *I prefer to work with a classmate than alone. It’s kind of relaxing to know that, if you don’t understand something, you can ask him/her at any time* (FG2_8); *Having Sam with me in mathematics class was great. We learned a lot together, and I feel really secure with him by my side* (FG1_9). In addition, they stated that they would like to have more peer tutoring experiences in future courses. *I would like to do more peer tutoring next year. You feel less stressed in class if you know that a colleague can help you* (FG2_7); *Working together is less stressful than doing it alone. I hope next year we do this in more subjects* (FG1_9). Regarding the second category, students seemed less anxious when being evaluated, as they had more trust in themselves. *The exam is the same, you know, but you trust yourself a little bit more if you see something you have explained before to someone. You think that if you explained it a week or two ago, you can do it now* (FG3_8). *I had explained a very similar problem to Jessica the week before. When I saw it in the exam, I knew I could do it and that she was going to be able to do it, too.* Having a peer that can help when the exam is close also seemed to have a positive effect on students’ evaluation anxiety. *I know I had Pete to help me with the exercises the days before the exam. Yeah, you can ask the teacher, but I prefer to ask him* (FG1_7). *I tried to do Ruffini for homework. No way. Then I was like chill, I’ll ask Allen tomorrow when we work in pairs, and then I’ll know how to do it for the exam* (FG8_9). In summary, students seemed to like the evaluation process being integrated into the peer tutoring process, as they did not find it as stressful.

## Discussion

The partial confirmation of hypothesis 1 (statistically significant differences will be reported between the pretest and the posttest for students in the experimental group in both, mathematics learning anxiety and mathematics evaluation anxiety and moderate effect sizes will be reported) was predictable, considering findings from previous research in the field. Recently, although not specifically in the field of mathematics, several authors, such as Knight et al. (2018) and Garba et al. (2019), documented anxiety improvements through peer tutoring in their respective fields of research. Consequently, it was not surprising that significant improvements were found. In addition, the qualitative information coming from the focus group sessions confirmed these improvements. Nevertheless, the rejection of this hypothesis due to the large effect size reported for mathematics learning anxiety (moderate effect sizes were expected) was not predictable (Hedge’s *g* = 0.84). Most meta-analyses and literature reviews in the field of peer tutoring in mathematics reported moderate effect sizes for these types of interventions in both psychological and academic outcomes (Bowman-Perrott et al., 2013, 2014; Alegre-Ansuategui et al., 2018). The effect size reported for mathematics evaluation anxiety (Hedge’s *g* = 0.42) is consistent and similar to findings previously reported in the field. Several authors have stated that mathematics evaluation anxiety is always greater and more difficult to address than mathematics learning anxiety (Ling, 2017; Yáñez-Marquina and Villardón-Gallego, 2017). As such, it was reasonable to find greater improvements for learning anxiety than for evaluation anxiety. Moreover, the qualitative information obtained from the focus groups also reinforced this statement, as students seemed to have experienced larger gains regarding learning than regarding evaluation. Nevertheless, the fact that effect sizes for one factor were double the effect sizes for the other (Hedge’s *g* = 0.84 vs Hedge’s *g* = 0.42) is not consistent with previous literature in the field and requires further examination in future research.

The confirmation of hypothesis 2 (posttest scores for the experimental group in both, mathematics learning anxiety and mathematics evaluation anxiety will be significantly lower than the posttest scores for the control group) was predictable taking into account the findings of recent studies in the field of peer tutoring and mathematics (Campbell, 2019; Grove et al., 2019; Moliner and Alegre, 2020; Yoo, 2020). In them, it is reported how the experimental group outscores the control group and statistically significantly differences are found when analyzing other psychological variables such as mathematics self-concepts or mathematics attitude. Hence, it could be expected that the posttest scores for the experimental group would be significantly better than the posttest scores for the control group.

The fact that hypothesis 3 was confirmed (no statistically significant differences will be reported for the posttest scores among 7th, 8th, and 9th grade students’ in the experimental group in mathematics learning anxiety or mathematics evaluation anxiety) is consistent with previous research in the field (Hill et al., 2016; Ramirez et al., 2018; Geary et al., 2019). According to these authors, the differences by gender regarding mathematics anxiety are more likely to appear during students’ high school years and college than during primary school or middle school. Analogously, the fact that hypothesis 4 (no statistically significant differences will be reported for the pretest or posttest scores between female and male students’ mathematics learning anxiety and mathematics evaluation anxiety) is also consistent with previous literature in the field. Authors such as Gresham and Burleigh (2019), Macmull and Ashkenazi (2019), and Morosanova et al. (2020) reported that, although mathematics anxiety increases through the years, differences are difficult to report within the same educational stage. That is, although important differences in mathematics anxiety may be reported between primary school, middle school, high school, and college students, students in middle school are likely to report similar results in mathematics anxiety independent of the course grade they are taking. In this sense and regarding hypotheses 3 and 4, several authors in the mathematics peer tutoring field have found no differences in academic or psychological outcomes by gender or course grade within the same educational stage (Alegre et al., 2019c; Hartini, 2019; McCurdy et al., 2020; Sun et al., 2020). The qualitative information supported these findings, as no important differences in students’ opinions were detected by gender or course grade. Most students seemed to have enjoyed the experience and reduced their mathematics anxiety levels independent of these two variables.

### Limitations

Although the potential positive impact of peer tutoring on middle school students’ mathematics anxiety seems quite evident considering the results reported in this research, certain limitations must be considered when interpreting them. First, the sample size, although not considered short or trivial by many researchers in the educational psychology field, cannot be considered large, either (Hendrickson et al., 2019; Sassenberg and Ditrich, 2019). Also, the sample was obtained by means of an intentional sampling (non-probabilistic) and only a single middle school participated in the study, so it is not representative of middle school students in Spain nor students outside the country. Moreover, as noted previously, this peer tutoring experience was designed to optimize the psychological outcome. Future research must test the effects of peer tutoring on mathematics anxiety under different circumstances (low or high sociocultural and socioeconomic status of the students’ families, lower or higher number of peer tutoring sessions, more or fewer months of implementation, more or less time for the tutoring interactions by session, as examples), as it may not be as effective as shown in this research (Funder and Ozer, 2019; Rutkowski et al., 2019). Furthermore, researchers of this manuscript, as stated above, did their best efforts to try to avoid a Hawthorne effect or similar and there is no evidence or record that something similar may have taken place during this research. Nevertheless, the possibility that experimental group students talked with control group students leading to a change in the conduct of some students and therefore to an alteration of the results in the study must be taken into account. Moreover, although the same teachers that taught students in the experimental group also taught in the control group, this study is not immune to the clustering effect, that is, the abilities, competence, experience and knowledge of the middle school teachers that participated in this research may have also influenced the outcome of the experience.

### Considerations for Future Research

It would have been interesting to test the simultaneous effects on students’ mathematics achievements and investigate the possible relationships between those factors. Unfortunately, it was impossible to obtain legal consent to include students’ mathematics marks in this research. The School Council only authorize the researchers of this article to measure and report students’ mathematics anxiety, but no permission was obtained to use any academic achievement variable or any related achievement index for this research. One of the main reasons we want to decrease mathematics anxiety is so that students will improve their mathematics achievement. The decrease in anxiety could just be in stated attitudes, with no performance-related change actually taking place. This must be considered as a possible future topic of research, as it is necessary to determine if the reported decreases in students’ mathematics anxiety correlated with an improvement in students’ mathematics achievements.

### Conclusion

The main conclusion that can be drawn from this study is that peer tutoring may be very beneficial for middle school students’ (12–15 years old) mathematics anxiety, independent of their gender or their course grade. Considering the results of this research, same-age and reciprocal peer tutoring is recommended for practitioners in the field who want to improve students’ mathematics anxiety. Additionally, from an organizational perspective, same-age and reciprocal tutoring are easier to carry out, as they may be implemented within the same classroom. The promising results of this research as well as of previous research in the field suggest no more than 20 min of interactions between pairs of students by session and no more than three tutoring sessions per week. Including a control group is highly recommended, as effect sizes may be overestimated due to its absence. Furthermore, in light of previous studies in the literature, practitioners in the field may find improvements not only in students’ mathematics anxiety, but also in other academic and psychological variables, such as self-concept or attitude toward mathematics. Students’ mathematics learning anxiety is expected to be lower and easier to reduce than students’ mathematics evaluation anxiety. Although the effect size for students’ mathematics learning anxiety was large in this research and future research is needed regarding this issue, effect sizes in these types of interventions are expected to be moderate, as was the case for mathematics evaluation anxiety. Although results may seem very promising, this research has important limitations (non-probabilistic sampling, quasi-experimental design, sample size…) that must be considered. Caution is required when interpreting the results as more evidence is needed to confirm the potential effects of peer tutoring on middle school students’ mathematics anxiety.

## Data Availability Statement

The raw data supporting the conclusions of this article will be made available by the authors, without undue reservation, to any qualified researcher.

## Ethics Statement

The studies involving human participants were reviewed and approved by the Valencian Autonomic Department of Education and Research Ethics Committee. The patients/participants provided their written informed consent to participate in this study.

## Author Contributions

LM was responsible for selecting the instruments, designing the intervention, and overseeing and editing the manuscript. FA was responsible for data collection, data analysis, and completing the first draft of the manuscript. All authors contributed to the article and approved the submitted version.

## Conflict of Interest

The authors declare that the research was conducted in the absence of any commercial or financial relationships that could be construed as a potential conflict of interest.
